# The Effects of Maternal Subclinical Hypothyroidism on Fetal Thymus Size: A Prospective Study

**DOI:** 10.3390/diagnostics15030276

**Published:** 2025-01-24

**Authors:** Mehmet Albayrak, Bekir Yükcü

**Affiliations:** 1 Perinatology Department, Giresun Obstetric and Pediatric Disease Education and Research Hospital, 28200 Giresun, Türkiye; 2 Pediatric Cardiology Department, Giresun Obstetric and Pediatric Disease Education and Research Hospital, 28200 Giresun, Türkiye; byukcu@gmail.com

**Keywords:** maternal subclinical hypothyroidism, fetal thymus, thyroid hormones, pregnancy, fetal development, thymus–thorax ratio

## Abstract

**Objective**: This study investigated the impact of maternal subclinical hypothyroidism on fetal thymus size and development and explored how inadequate thyroid hormone production in pregnant women affects the fetal thymus. **Methods**: Conducted at the Giresun Obstetrics, Gynecology, and Pediatrics Training and Research Hospital, this case–control study involved 86 pregnant women, 43 with hypothyroidism and 43 without. Maternal thyroid function was assessed using TSH and free T4 levels, and fetal thymus size and thymus–thorax ratio were measured using ultrasound. Exclusion criteria were chronic hypertension, gestational hypertension or eclampsia, multiple pregnancies, infectious diseases, renovascular diseases, diagnosed with hypothyroidism prior to pregnancy and other endocrine disorders, fetal cardiac diseases, and morbid obesity. Data collected included maternal age, gestational week, number of pregnancies, parity, number of living children, thyroid-stimulating hormone (TSH) and Free thyroxine 4 (T4) levels, and fetal thymus measurements (transverse diameter and thymus/thorax ratio). Statistical analyses were performed using the Mann–Whitney U test and logistic regression analysis. The relationships between TSH, thymus diameters, thorax diameters, and the thymus–thorax ratio were evaluated using Spearman’s correlation coefficient. **Results**: The thymus–thorax ratio was significantly reduced in the hypothyroid group (*p* = 0.003). Logistic regression analysis identified TSH as an independent risk factor for a low thymus–thorax ratio, with each unit increase in TSH associated with a 1.345-fold higher likelihood of having a low thymus–thorax ratio. A significant negative correlation was found between TSH levels and the TTR ratio (Spearman’s correlation coefficient r = −0.338, *p* = 0.001). **Conclusions**: An association was identified between maternal TSH levels and the thymus–thorax ratio, with increasing TSH levels correlating with a decrease in the thymus–thorax ratio. Regular monitoring of thyroid hormone levels during pregnancy and appropriate replacement treatment in cases of deficiency are crucial for optimal fetal thymus development. Further multicenter studies are needed to confirm these findings and investigate the long-term implications of altered fetal thymus development.

## 1. Introduction

The thyroid gland generates hormones that are necessary for healthy development and metabolism, particularly during pregnancy when the body needs more thyroid hormones. The main cause of hypothyroidism is insufficient thyroid hormone secretion. Free thyroxine 4 (FT4) concentrations below the standard range and elevated thyroid-stimulating hormone (TSH) levels are the hallmarks of primary hypothyroidism [1]. Hypothyroidism can be caused by a variety of conditions, such as iodine deficiency, Hashimoto thyroiditis [2] and uncommon causes, including congenital, drug-related, iatrogenic, and infiltrative illnesses [1].

Hypothyroidism is one of the most frequent chronic disorders during pregnancy [3,4]. Pregnant women may be affected by hormonal changes during pregnancy. Several factors can affect the thyroid function of mothers, including elevated levels of human chorionic gonadotropin, increased synthesis and secretion of thyroxine-binding globulin by the liver, and increased renal clearance of iodine. In many patients with hypothyroidism, the absence of overt clinical symptoms is referred to as subclinical hypothyroidism [5]. With a prevalence ranging from 0.5% to 3.47% throughout pregnancy [3,4].

Undiagnosed and untreated maternal hypothyroidism raises concerns about pregnancy problems [1]. The normal function of the placenta during pregnancy depends on thyroid hormones, and a lack of these hormones can result in placental dysfunction [6]. Maternal hypothyroidism increases the risk of pregnancy complications such as gestational hypertension, pre-eclampsia [7], gestational diabetes, and preterm labor [8,9,10]. The therapy of minor variations in maternal thyroid function is up for debate, but it is well-accepted that severe hypothyroidism should be treated to avoid difficulties for both the mother and the fetus [1,11,12,13,14].

Hypothyroidism or insufficient thyroid hormone production can lead to serious complications not only in the mother but also in the fetus. A deficiency in thyroid hormones during pregnancy can negatively affect the normal growth and developmental processes of the fetus. This condition has been associated with adverse outcomes such as intrauterine growth restriction, placental dysfunction, low birth weight, an increased risk of preterm birth, and impaired neurodevelopment [15].

The thymus is a major lymphoid organ that provides protection and surveillance for the body’s defense mechanism. T-cell lymphocyte development, differentiation, and selection are all significantly affected by it [16]. The thymus begins to develop in the fifth week of pregnancy from the third pharyngeal sac (ventral portion), and in the ninth week, lymphocytes begin to form in the thymus. From the time of pregnancy until puberty, the thymus grows and increases continually [17]. The thymus is morphologically complete at 16–20 gestational weeks. Pregnancy-related thymic involution can happen in response to several acute stressors, including illness, trauma, sepsis, and malnourishment. It is triggered by systemic inflammatory mediators and endogenous corticosteroids in response to the aforementioned circumstances. These disorders are also the main causes of fetal growth restriction (FGR) [18]. Moreover, FGR increases the risk of fetal morbidity and mortality; therefore, it may indirectly affect the size of the fetal thymus [18].

Fetal thymus size is a sensitive predictive tool for pregnancy-related conditions, such as chorioamnionitis with or without premature rupture of the membrane [19], eclampsia [20], preterm labor [21], and gestational diabetes, according to recent research [22].

Previous studies have highlighted the association between maternal thyroid dysfunction and various fetal developmental issues [15,16]. The effects of maternal hypothyroidism on the embryonic immune system and the development of the fetal thymus are still poorly understood despite the awareness of these dangers. Understanding this relationship is crucial for developing targeted interventions to mitigate the adverse effects of hypothyroidism on fetal health.

This study aimed to fill this gap by investigating the effects of maternal hypothyroidism on fetal thymus size. By analyzing data from pregnant women diagnosed with hypothyroidism and comparing them with a control group of euthyroid pregnant women, this study sought to elucidate the extent to which hypothyroidism affects fetal thymus development.

## 2. Material and Methods

### 2.1. Study Population

This study was conducted at the Giresun Obstetrics and Pediatrics Training and Research Hospital and included patients attending obstetrics, perinatology, and pediatric cardiology outpatient clinics for fetal monitoring. The participants were women aged 20–42 years who visited the clinic for routine antenatal checkups during their second trimester (18–24 weeks of gestation) and were screened for fetal anomalies.

At the initial evaluation, demographic and clinical data were collected, including age, gravidity, parity, body mass index (BMI), and sonographic measurements of fetal biometry, such as biparietal diameter (BPD), abdominal circumference (AC), femur length (FL), and estimated fetal weight (EFW). Gestational age was confirmed using the first-trimester ultrasound data. The recorded variables included maternal age, sex, marital status, hemoglobin (Hb), blood glucose, thyroid-stimulating hormone (TSH), free T4, aspartate aminotransferase (AST), alanine aminotransferase (ALT), blood urea nitrogen (BUN), and creatinine.

Participants in the second trimester who underwent routine second-level ultrasonography were included if they met the criteria for hypothyroidism. The updated ATA guidelines published in 2017 recommend using a laboratory- or population-based pregnancy-specific TSH reference range. In cases where such a reference range is unavailable, the guidelines suggest an upper normal limit cut-off of 4.0 mIU/L for TSH, which typically represents a reduction of approximately 0.5 mIU/L compared to the non-pregnant TSH reference range. However, in our region, the TSH reference range for second-trimester pregnant women has been established as 0.35–4.1 mIU/mL. Based on this reference range, 43 patients were identified as euthyroid, while 43 patients with elevated TSH levels and normal FT4 levels were diagnosed with subclinical hypothyroidism [1]. These patients were subsequently referred to a local endocrinologist for further evaluation, and levothyroxine therapy was initiated for those deemed necessary.

The exclusion criteria are as follows:

- Patients with a systemic disease;
- Multiple pregnancies and gestations, invitro fertilization;
- Smoking;
- Biochemical, hormonal, or hematological abnormalities;
- Congenital fetal anomalies (cardiac, liver, neurological, etc.);
- Gestational diabetes;
- Chronic hypertension;
- Gestational hypertension or eclampsia;
- Acute and chronic infections (fever, urinary tract infections, hepatitis or others);
- Renovascular diseases;
- Maternal morbid obesity;
- Diagnosed with hypothyroidism prior to pregnancy and other endocrine disorders.

**Figure 1:**
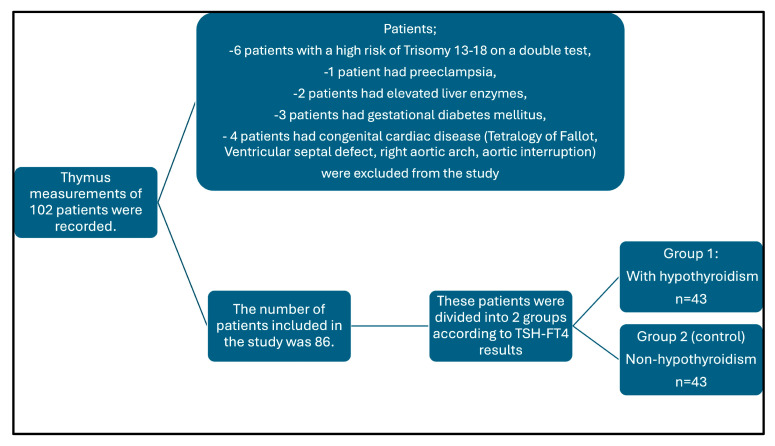
Flowchart of included and excluded patients in the study.

A total of 102 patients who underwent thymus imaging and had their data recorded were initially included in the study, considering the previously mentioned exclusion criteria. However, 16 of these patients were excluded from the study for the following reasons: six patients had a high risk of trisomy 13–18 based on a double test, one patient had pre-eclampsia, two patients had elevated liver enzymes, three patients were diagnosed with gestational diabetes mellitus, and four patients had congenital cardiac diseases (Tetralogy of Fallot, ventricular septal defect, right aortic arch, and aortic interruption). Consequently, data from 86 patients were analyzed (Figure 1).

### 2.2. Fetal Thymus İmage Analysis

The fetal thymus size (transverse diameter and anteroposterior diameter), thorax size (thorax anteroposterior diameter), and thymus–thorax ratio were recorded by an experienced perinatologist and a pediatric cardiologist. All measurements were performed using the Mindray Resona R9 device (Mindray Bio-Medical Electronics Co., Ltd., Shenzhen, China). The thymus images of the fetuses were first recorded on the ultrasound device, and then both doctors evaluated these images and entered the measurement results into the dataset with a final decision.

**Figure 2:**
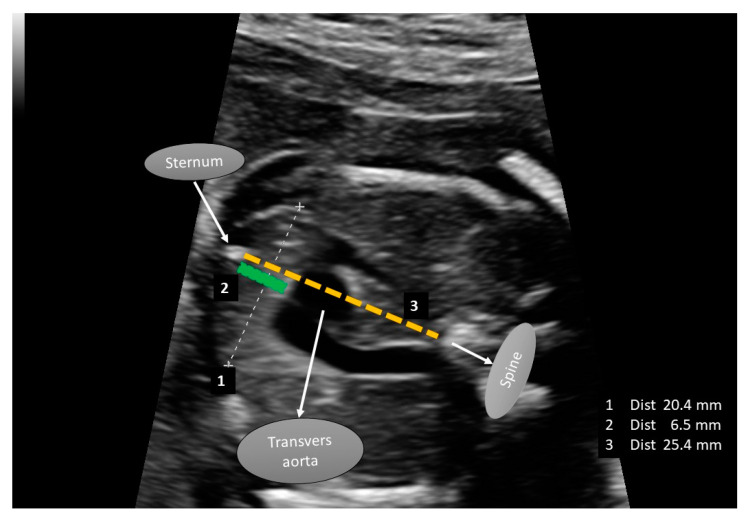
Fetal thymus measurements: The figure illustrates the measurements obtained during the evaluation of the fetal thymus. The measurement labeled as 1, represented by the white dashed line, indicates the fetal thymus transverse diameter. The measurement labeled as 2, represented by the green solid line, corresponds to the fetal thymus anteroposterior diameter. The measurement labeled as 3, represented by the yellow dashed line, denotes the thorax anteroposterior diameter.

During the ultrasonographic assessment, the patient was placed in the supine position on the examination table. This was measured when the fetus was inactive. The thymus–thorax ratio (TTR) was determined by measuring the thymic and thoracic diameters perpendicular to the line connecting the centers of the sternum and the thoracic spine [23] (Figure 2). The anteroposterior diameter of the fetal thymus was measured along the midline, extending from the posterior border of the sternum to the anterior border of the transverse aortic arch. Similarly, the anteroposterior mediastinal diameter of the thorax was measured along the same line, extending from the posterior border of the sternum to the anterior border of the thoracic spine. Additionally, the left brachiocephalic vein (LBCV), located in a different plane, can serve as an additional anatomical landmark to define the posterior border of the thymus. In previous studies, the intrathymic course of the LBCV has been associated with congenital heart diseases (CHDs), and therefore, special attention was given to this structure [24]. The TTR was calculated as the ratio of the anteroposterior thymic diameter to the anteroposterior mediastinal thoracic diameter [25,26,27] (Figure 2). These measurements had no impact on the patient’s follow-up or treatment.

### 2.3. TSH and Free T4 Measurement

Venous blood samples were collected using BD Vacutainer SST II Advance tubes. The samples were centrifuged at 1500× *g* for 10 min to obtain serum. Thyroid-stimulating hormone (TSH) and free T4 levels were measured using a Roche Cobas e601 analyzer (Roche Diagnostics GmbH, Mannheim, Germany).

### 2.4. Hypothesis

The hypothesis of this study was that maternal hypothyroidism may negatively impact fetal thymus size and development. Maternal hypothyroidism is characterized by inadequate thyroid hormone production, which may adversely affect fetal development, particularly the growth and function of the fetal thymus, which depends on thyroid hormones.

### 2.5. Statistical Analyses

Statistical analyses were conducted using IBM SPSS Statistics version 25 to evaluate the findings of the study. Descriptive statistics, including the median (25th and 75th percentiles), were used for quantitative data, as the data were not normally distributed based on the Shapiro–Wilk test results. For qualitative data, frequency, and percentage values were presented.

The Mann–Whitney U test was applied to compare non-normally distributed quantitative variables between the two groups. Spearman’s correlation coefficient was used to assess the relationships between variables. Logistic regression analysis was performed to evaluate the effects of risk factors on the low thymus–thorax ratio. For comparisons of categorical variables, Fisher’s exact test was utilized. To predict hypothyroidism based on the thymus–thorax ratio, ROC Curve analysis and diagnostic screening tests were conducted. Statistical significance was set at *p* < 0.05, and the results were reported with 95% confidence intervals.

### 2.6. Ethic Consideration

This study was approved by the Giresun Education and Research Ethical Committee (protocol code 03 and date 13 June 2024). Written informed consent was obtained from all study participants in accordance with the Declaration of Helsinki Principles, as revised in 2008.

## 3. Results

The study was conducted with 86 female patients at the Giresun Obstetrics, and Pediatrics Training and Research Hospital. The ages of the patients ranged between 20 and 42 years, with a median (25th and 75th percentiles) of 28 (25.75–34) years. The gestational age of the cases ranged between 17 and 25 weeks, and the median (25th and 75th percentiles) was 22 (21–22) (Table 1).

Among the participants, 50% (*n* = 43) were diagnosed with hypothyroidism and 50% (*n* = 43) were not (Table 2).

There were no statistically significant differences between the groups in terms of age, thymus transverse diameter, gestational week, T4 level, and EFW (*p* > 0.05). While there was no significant difference between the groups in terms of the number of pregnancies (*p* = 0.187), significant differences were observed in parity and the number of living children. The proportion of individuals who had never given birth was higher in the hypothyroidism group (*p* = 0.009), and the proportion of those with only one child was significantly greater (*p* = 0.012).

The thymus/thorax ratio in the non-hypothyroid group was significantly higher than that in the hypothyroid group (*p* = 0.003).

**Figure 3:**
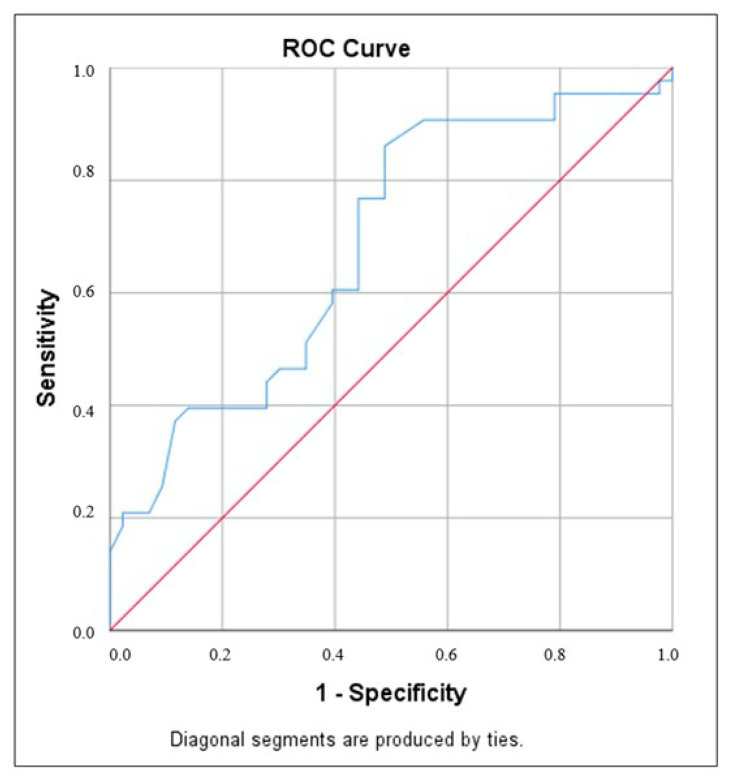
ROC curve for thymus/thorax ratio and hypothyroidism. The blue line represents the TTR (thymus–thorax ratio), while the red line indicates the reference point. The ROC curve illustrates the diagnostic performance of the TTR in predicting hypothyroidism. Abbreviation: ROC—Receiver Operating Characteristic.

In our study, a cut-off value of 0.345 for the correlation between the thymus/thorax ratio and the presence of hypothyroidism demonstrated a sensitivity of 60.5% and a specificity of 60.5%. The area under the ROC curve was 0.684, with a standard error of 5.8%. A statistically significant relationship was observed between the thymus/thorax ratio cut-off value of 0.345 and the presence of hypothyroidism (*p* = 0.003) (Table 3, Figure 3).

### 3.1. Logistic Regression Analysis

Logistic regression analysis was performed with potential independent variables such as TSH, parity, and number of living children that could influence a low thymus–thorax ratio (Table 4).

The variables included in the study were evaluated using logistic regression analysis. After the second step, TSH formed a significant model for the risk factors affecting low TTR (*p* < 0.05). The explanatory coefficient of the model was 64%. Logistic regression identified TSH as an independent risk factor for a low thymus–thorax ratio, with each unit increase in TSH associated with a 1.345-fold higher likelihood of having a low thymus–thorax ratio.

### 3.2. Spearman’s Correlation Coefficient

The relationship between TSH levels and the thymus–thorax diameters and ratio was evaluated using Spearman’s correlation coefficient. No statistically significant correlation was found between TSH levels and the thymus transverse, thymus anteroposterior, or thorax anteroposterior diameters. However, a significant negative correlation was found between TSH levels and the TTR ratio (r = −0.338, *p* = 0.001). This suggests that an increase in TSH levels tends to decrease the TTR ratio (Table 5).

## 4. Discussion

This study explored the effects of maternal hypothyroidism on fetal thymus size, contributing to the growing body of evidence linking maternal thyroid dysfunction with adverse pregnancy outcomes and fetal development issues. Our findings indicate that there is a significant relationship between maternal hypothyroidism and the thymus–thorax ratio. A negative association was identified between maternal TSH levels and the thymus–thorax ratio, with increasing TSH levels correlating with a decrease in the thymus–thorax ratio. These results align with the existing literature on the role of maternal thyroid function in fetal thymus development. However, in contrast to the literature and our findings, Kasap et al. [27] reported an increased fetal thymic-thoracic ratio (TTR) in pregnant women with Hashimoto’s disease. These discrepancies in the literature suggest that the effects of thyroid disorders on the thymus have not yet been fully elucidated and that other complex mechanisms may also influence thymus development.

The most common cause of hypothyroidism is Hashimoto thyroiditis, an autoimmune condition. During pregnancy, hypothyroidism may arise due to several factors, including the onset of Hashimoto’s thyroiditis, insufficient treatment of pre-existing hypothyroidism, or overtreatment of hyperthyroidism with anti-thyroid medications. Approximately 2.5% of pregnant women experience mildly elevated thyroid-stimulating hormone (TSH) levels above 6 mIU/L, while 0.4% have significantly elevated levels exceeding 10 mIU/L [28]. Untreated or poorly managed hypothyroidism during pregnancy significantly increases the risk of miscarriage and maternal complications such as anemia, muscle weakness, heart failure, pre-eclampsia, placental abnormalities, and postpartum hemorrhage [28]. These risks are more pronounced in severe cases of hypothyroidism and may also be elevated in women with thyroid peroxidase (TPO) antibodies. Mild hypothyroidism often goes unnoticed, as symptoms can be subtle or mistaken for typical pregnancy-related changes.

In the first 18–20 weeks of pregnancy, the developing fetus relies entirely on the mother for thyroid hormone production. By mid-pregnancy, the fetus’s thyroid gland becomes functional and starts producing hormones independently [2].

The impact of hypothyroidism on the fetus has been extensively studied in animal models, particularly in rodents. These studies clearly demonstrated the detrimental effects of hypothyroidism on neural development and other endocrine systems. Research in thyroidectomized fetal sheep further highlights how hypothyroidism alters muscle composition and function as well as bone development and ossification. These findings are consistent with clinical observations in humans, where fetal hypothyroidism can lead to mental retardation, delayed bone development, and impaired growth [2].

The thymus gland, a primary lymphoid organ, is where T cells, essential for the immune system, complete their development. Previous studies have linked alterations in thymus size, such as hypoplasia or hyperplasia, with various diseases, including infectious diseases, aneuploidies, endocrine disorders, and genetic syndromes [29]. For instance, a small thymus size has been associated with fetal growth restriction, gestational diabetes, chorioamnionitis, pre-eclampsia, 22q11.2 deletion syndrome, and rare genetic syndromes such as Ellis–Van Creveld syndrome and chondrodysplasia punctata [30,31,32,33,34]. Fetal thymus size has also been investigated in the context of fetal inflammatory response syndrome, which is associated with various pathological conditions, including intrauterine infections and inflammation [29,30]. Reduced thymus size has been linked to these inflammatory conditions, suggesting compromised immune system development [29]. In certain cases, an increase in fetal thymus size has been observed. Gasthaus et al. reported that maternal HIV infection was associated with significantly larger fetal thymus size in their study examining the impact of maternal HIV infection on thymic development. Similarly, another study demonstrated increased thymus size in fetuses diagnosed as small for gestational age [34,35].

A study has also shown that low vitamin D levels can lead to a reduction in thymus size [36]. However, since vitamin D levels were not included in our study design, we were unable to assess their impact on our patients.

Our findings are consistent with those of previous research on fetal thymus size in pregnancies complicated by maternal conditions. Gok et al. [37] found that the fetal thymus size was significantly reduced in pregnancies complicated by maternal diabetes. Similarly, Ghalandarpoor-Attar et al. [38] found that pregnant women with diabetes had smaller fetal thymus sizes than non-diabetic pregnant women. They further suggested that measuring fetal thymus size could serve as a useful method for screening for diabetes during pregnancy. In contrast, Kasap et al. reported an increased fetal thymic-thoracic ratio in pregnant women with Hashimoto’s disease, suggesting that the nature of maternal thyroid dysfunction can affect fetal thymus development [27].

Recent studies have highlighted a significant link between early maternal thyroid function and fetal growth, underscoring the essential role of maternal thyroid hormones in normal fetal development [2,3,4,5,6,7,8,9]. Furthermore, Cromi et al. [18] and Tong et al. [39] explored the connection between fetal thymus size and growth restriction due to placental insufficiency, reinforcing the idea that reduced fetal thymus size is strongly associated with fetal growth restriction. One of the significant findings of our study was the association between maternal hypothyroidism and smaller fetal thymus size, which may suggest a potential negative impact on fetal growth restriction and other growth-related parameters. However, as these parameters were not assessed in our study, no definitive conclusions could be drawn. In our study, considering the observed reduction in fetal thymus size in pregnant women with hypothyroidism, we suggest a potential link between thyroid function and fetal immune development, which indirectly supports these findings.

Therefore, during fetal anomaly screening, it is important to evaluate the fetal thymus with particular attention to the TTR. In cases of low TTR, other parameters that may affect fetal thymus development should be assessed, and maternal thyroid function tests should also be included in the evaluation. Additionally, in cases of maternal hypothyroidism, fetal thymus development and size should be carefully considered as part of the overall assessment.

### Study Limitations

The observational design, relatively small sample size, and lack of data on the long-term impact of altered fetal thymus development on postnatal immune function are notable limitations of this study. Future research should focus on larger multicenter studies to confirm these findings and explore the underlying mechanisms by which maternal thyroid function affects fetal thymus development. Additionally, longitudinal studies are needed to assess the long-term impact of altered fetal thymus development on postnatal immune function and evaluate the potential benefits of various thyroid hormone replacement therapies. Specifically, research should examine the timing and dosage of thyroid hormone supplementation to prevent adverse effects of maternal hypothyroidism on fetal thymus development. Such investigations could provide valuable insights into clinical practice. Also, in our study, all patients in the hypothyroidism group had subclinical hypothyroidism. Due to the insufficient number of patients with overt hypothyroidism, this comparison could not be performed, which represents one of the limitations of our study. Future studies are recommended to examine not only the effects of subclinical hypothyroidism on fetal thymus size but also to compare these effects with those of overt hypothyroidism.

## 5. Conclusions

This study provides significant evidence that maternal subclinical hypothyroidism adversely affects the fetal thymus size. These findings highlight the importance of maintaining optimal thyroid function during pregnancy to ensure proper development of the fetal immune system. Continued research is necessary to fully understand the long-term consequences and develop targeted interventions to support fetal development in hypothyroid pregnancies.

## Figures and Tables

**Table 1:** Distribution of Participants’ Demographic and Clinical Characteristics.

Characteristics		*n* (%) Median (25th and 75th Percentiles)
Age (years)		28 (25.75–34)
Thymus transverse diameter (mm)		18 (16–20)
Thymus/thorax ratio		0.345 (0.290–0.390)
Gestational week (week)		22 (21–22)
Number of pregnancies (no)	1	35 (40.7)
	2	20 (23.3)
	≥3	31 (36)
Parity (no)	0	36 (41.9)
	1	37 (43)
	2	12 (14)
	≥3	1 (1.2)
Number of living children (no)	1	38 (44.2)
	≥2	48 (55.8)
TSH (mIU/mL)		3.88 (1.58–4.52)
T4 (ng/dL)		1.17 (1–1.36)
EFW (gr)		467 (390–530)
Groups	No hypothyroidism	43 (50)
	Hypothyroidism	43 (50)

Abbreviations: TSH—Thyroid-Stimulating Hormone, T4—Free Thyroxine, EFW—Estimated Fetal Weight.

**Table 2:** Comparison of descriptive characteristics by groups.

Characteristics		Groups		*p*
		No Hypothyroidism (*n* = 43) Median (25th and 75th Percentiles) or *n* (%)	Hypothyroidism (*n* = 43) Median (25th and 75th Percentiles) or *n* (%)	
Age (years)		29 (26–33)	27 (25–34)	^a^ 0.634
Thymus transverse diameter (mm)		18 (16–21)	18 (16–19)	^a^ 0.240
Thymus/thorax ratio		0.375 (0.300–0.400)	0.320 (0.280, 0.350)	^a^ 0.003 *
Gestational week		22 (21–22)	22 (21–22)	^a^ 0.332
Number of Pregnancies (no)	1	18 (41.9)	17 (39.5)	^b^ 0.187
	2	6 (14)	14 (32.6)	
	≥3	19 (44.1)	12 (27.9)	
Parity (no)	0	12 (27.9)	24 (55.8)	^b^ 0.009 *
	1	20 (46.5)	17 (39.5)	
	2	10 (23.3)	2 (4.7)	
	≥3	1 (2.3)	0	
Number of living children (no)	1	12 (27.9)	26 (60.5)	^b^ 0.012 *
	≥2	31 (72.1)	17 (29.5)	
TSH (mIU/mL)		1.6 (1.23–2.28)	4.5 (4.28–4.90)	^a^ <0.001 *
T4 (ng/dL)		1.21 (1–1.46)	1.17 (0.9–1.27)	^a^ 0.216
EFW (gr)		450 (377–530)	490 (419–529.5)	^a^ 0.410

The statistical tests used in the table are as follows: ^a^: Mann–Whitney U test; ^b^: Fisher Exact test. The symbols indicate significance levels: * *p* < 0.05. Abbreviations: TSH—Thyroid-Stimulating Hormone, T4—Free Thyroxine, EFW—Estimated Fetal Weight.

**Table 3:** Diagnostic Screening Tests and ROC Curve Results for Thymus/Thorax in Predicting the Presence of Hypothyroidism.

Parameter	ROC Curve					*p*
	Cutoff	Sensitivity	Specificity	Area	95% Confidence Interval	
Thymus/Thorax Ratio	≤0.345	60.5%	60.5%	0.684	0.571–0.798	0.003 *

Abbreviation: ROC—Receiver Operating Characteristic. The symbol indicates significance levels: * represents *p* < 0.05.

**Table 4:** Logistic Regression Analysis of Risk Factors Affecting Low Thymus–Thorax Ratio.

Parameter	*p*	ODDS Ratio	95% Confidence Interval	
			Lower	Upper
TSH	0.02 *	1.345	1.047	1.728

The symbol indicates significance levels: * represents *p* < 0.05. Abbreviations: TSH—Thyroid-Stimulating Hormone.

**Table 5:** Spearman’s correlation coefficient between TSH measurement and Thymus–Thorax Dimensions.

Parameters	TSH	
	*r*	*p*
Thymus transverse diameter (mm)	−0.085	0.435
Thymus AP diameter (mm)	−0.136	0.213
Thorax AP diameter (mm)	−0.007	0.947
Thymus/Thorax Ratio	−0.338	0.001 *

TSH and Thymus–Thorax diameters and the ratio of the relationship between variables were evaluated by Spearman’s correlation coefficient. The symbol indicates significance levels: * represents *p* < 0.05. Abbreviations: AP—Antero-posterior, TSH—Thyroid-Stimulating Hormone.

## Data Availability

The datasets used and/or analyzed during the current study are available from the corresponding author upon reasonable request.
